# Analysis of urgent inpatient neurologic consultations in a large tertiary hospital center: Follow‐up on the effect of standardized training of residents

**DOI:** 10.1002/brb3.2983

**Published:** 2023-03-27

**Authors:** Jiafang Wang, Min Ren, Hong Wang, Zhenzhen Bai, Kebin Zeng

**Affiliations:** ^1^ Department of Neurology First Affiliated Hospital of Chongqing Medical University Chongqing China; ^2^ Department of Neurology Affiliated Hospital of Chongqing Three Gorges Medical College Chongqing China; ^3^ Department of Neurology People's Hospital of Shapingba District Chongqing China; ^4^ Finance Department First Affiliated Hospital of Chongqing Medical University Chongqing China

**Keywords:** clinical analysis, multidisciplinary, neurological disorders, resident, urgent consultation

## Abstract

**Background:**

Clinical neurology is difficult for young residents. To familiarize with neurological emergencies as soon as possible for young doctors, the urgent inpatient neurologic consultations were analyzed.

**Methods:**

A retrospective study was conducted on the urgent inpatient neurologic consultations in a large tertiary hospital for 4 consecutive years.

**Results:**

A total of 1437 cases were included, and the annual consultation cases gradually decreased from 573 to 257, involving 29 clinical departments. The disorders of urgent inpatient neurologic consultations were divided into three categories: neurological disorders (77.8%), non‐neurological disorders (10.4%), and undiagnosed disorders (11.8%), common causes in consultation were disturbance of consciousness (36.0%), convulsions/stiffness (13.6%), limb weakness (8%), and mental disorder (5.6%). Common neurological disorders included acute cerebrovascular disease (33.6%), epilepsy/status epilepticus (15.8%), and metabolic or infectious toxic encephalopathy (14.9%).

**Conclusion:**

Urgent inpatient neurologic consultations involve multidisciplinary critical diseases, mainly neurological diseases. The standardized training of residents may help to rapidly improve the comprehensive diagnosis and treatment ability of young residents and is suitable for use in hospitals at all levels.

## Graphical abstract

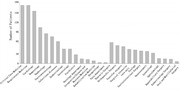

## 1 INTRODUCTION

Neurology is a branch of medicine that deals with disorders of the nervous system, including the central and peripheral nervous systems. Neurological practice relies heavily on knowledge of neuroscience and the neuroanatomy; thus, most people recognize that neurology is a complex and difficult subject (Zhao et al., 2020). According to one survey, difficulty understanding neurology is common among not only medical students but also older physicians (Chirra et al., 2019; Zhao et al., 2020), which presents some challenges for non‐neurologists, especially for young physicians (Dall et al., 2013; Zinchuk et al., 2010). For most physicians, neurology is a complex discipline that covers many diseases. It also brings many blind spots to the medical treatment of residents in different departments. Therefore, the urgent inpatient neurologic consultations are very common in tertiary hospital centers.

Acute and critical neurological disorders are characterized by rapid onset and rapid progression. If the disease is not properly diagnosed and treated promptly, there will be more complications and higher mortality. Rapid and accurate diagnosis and treatment are critical for improving outcomes in acute neurological disorders (Greene, 2018). The main purpose of the urgent inpatient neurologic consultations is to help other departments manage life‐threatening symptoms as quickly as possible. With rapid response, cure rates and quality of life are improved, whereas morbidity and mortality are reduced. After their training period in Spain, neurologists in training were informed about EEG in 49% of education units. It has been reported that the demand for neurology specialists exceeds the supply (Busetto et al., 2022; Rodríguez‐Antigüedad et al., 2011). However, there have been few previous studies that have looked into urgent inpatient neurologic consultations. The National Standardized Training of Residents was officially implemented in 2014. The effect of Standardized Resident Training is unknown. Thus, the purpose of this study is to look into urgent inpatient neurologic consultations in order to help young doimpactctors become familiar with neurological emergencies and to understand the effect of Standardized Training of Residents.

## 2 METHODS

### 2.1 Subjects

This study included clinical cases of non‐neurological inpatients who applied for urgent inpatient neurologic consultations in a 3200‐bed tertiary university hospital center at the First Affiliated Hospital of Chongqing Medical University, China, from January 1, 2014 to December 31, 2017. The study protocol was approved by the Ethics Committee of Chongqing Medical University.

### 2.2 Methods

Urgent inpatient neurologic consultations require a non‐neurologist physician to contact the neurology physician by phone first and then complete the electronic case request form, including patient name, age, gender, admission number, bed number, consulting specialty, and reasons for the consultation. The neurologic consultants should be at the applicant's ward within 10 min of receiving the call. Urgent consultations are usually handled by the chief neurologic residents through Monday to Friday, and by the attending physicians on weekends and holidays. For difficult cases, the young physician consults the senior attending on duty.

After reviewing the patient's information, the neurologists reviews the medical history, performs neurological examination, makes a clinical diagnosis, and recommends further examinations and treatment plans. For patients who had multiple neuroconsultations, this study regarded the conclusion of the last consultation as the final diagnosis. Diagnoses of non‐neurological disorders were made from the patient's discharge record. If the same case is referred more than twice, it is still regarded as only one case. The following parameters were analyzed: purpose of consultation, spectrum of consultation diseases, departments distribution of non‐neurological disease, and compliance with urgent inpatient neurologic consultations criteria. There were no reported criteria for urgent inpatient neurologic consultations; therefore, we defined the criteria as follows (Minardi et al., 2019; Zhao et al., 2020): (1) stroke within 24 h; (2) cases of critical illness requiring the assistance of neurological physicians; (3) acute gas poisoning within 24 h requiring emergency hyperbaric oxygen treatment; (4) other newly emerging neurological disease symptoms, neurological signs, or imaging abnormalities; (5) patients to receive emergency surgery with neurological diseases previously for preoperative evaluation; and (6) cases with medical disputes.

### 2.3 Statistical analysis

Microsoft Excel was used for data collection and to create tables. Countable data values were presented as the mean ± SD. Data processing was done using SPSS version 23.0.

## 3 RESULT

### 3.1 General information

There were 1618 urgent inpatient neurologic consultations within 4 years: 573 in 2014, 406 in 2015, 382 in 2016, and 257 in 2017, which decreased year after year. Of these, 136 had at least two consultations, these were each considered 1 case in the data, and 1437 were eventually included in the study, of whom 54.6% were male and 45.4% were female, with a mean age of 60.3 years (range: 9–103).

### 3.2 Consulting departments involved

**FIGURE 1:**
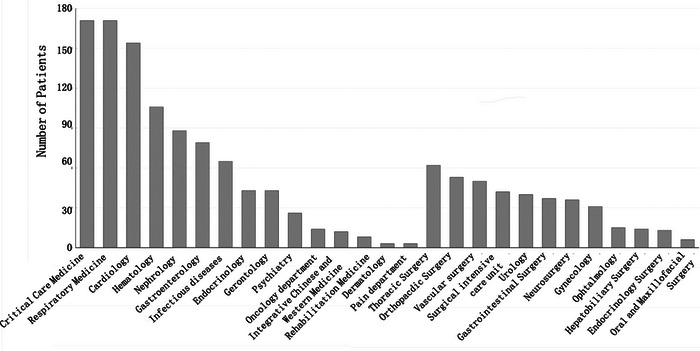
Department distribution of urgent inpatient neurologic consultations.

Urgent inpatient neurologic consultations involved 29 clinical departments (Figure 1), 990 cases in the general ward and 447 cases in the intensive care unit (ICU). Overall, 68.3% of cases were from internal medicine and surgery, which was similar with the observations previously in Jordan (Lahlouh et al., 2020). Among the internal medicine departments, the departments that applied for urgent consultations were the emergency department (12%), respiratory department (11.9%), cardiology department (11%), hematology department (7%), and nephrology department (6%). The departments that requested the most urgent consultations among the surgical departments were thoracic surgery (13%), orthopedics (12%), vascular surgery (11%), hepatobiliary surgery (10%), and ICU surgery (10%).

### 3.3 Illness and counseling purposes

Common purposes for urgent inpatient neurologic consultations were the undiagnosed diseases (80%), assistance in treatment (14%), and controversial cases (6%) (Table 1). Table 1 also shows less common reasons for requesting urgent inpatient neurologic consultations.

**TABLE 1:** Reasons for urgent inpatient neurologic consultations.

Reasons for consultation	Number of cases
Altered consciousness	517
Hyperspasmia/rigidity	195
Extremity weakness	116
Mental disturbance	81
Department transference	69
Headache	66
Adjustment of treatment plan	58
Dysarthria	43
Dizziness	25
Aphasia	27
Emesis	24
Operative risk assessment	23
Involuntary movement	22
Head imaging abnormalities	16
Numbness and pain	10
Lumbar puncture	9
Thrombolysis therapy	9
Hyperbaric oxygen therapy	7
Cognitive impairment	7
Anisocoria	6
Vision loss	5
Positive sign of meninges stimulation	3
Requirements of the family	3
Blepharoptosis	2
Diplopia	1
Intrathecal administration for drugs	1
Increased heart rate	1
Emergency head MRI	1

**FIGURE 2:**
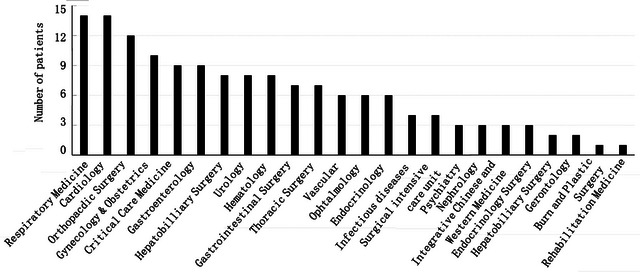
Nonnervous system diseases distribution in urgent inpatient neurologic consultations.

Diseases for urgent inpatient neurologic consultations can be divided into three categories: neurological diseases, non‐neurological diseases, and unknown diseases. Conditions that frequently required neurological consultations included acute cerebrovascular disease, epilepsy, intracranial infection, metabolic encephalopathy, infectious toxic encephalopathy, and hypoxic ischemic encephalopathy; these accounted for 58.4% of total urgent inpatient neurologic consultations. The patients’ requests more frequently for urgent inpatient neurologic consultations in the older patients with mean age 74.5 years in the study. Non‐neurological diseases accounted for 10.4% of the total urgent neurologic consultations. Some of the cases were used to rule out potential problems during the differential diagnosis process. However, some of them were not. It implies that they lack both the necessary standardized training and basic medical knowledge. Unknown diseases accounted for 11.8%. There were many reasons for these, including highly complicated diseases and refusal of further testing by the patient (Figure 2).

## 4 DISCUSSION

The study obtained two main findings: First, urgent inpatient neurologic consultations involve multidisciplinary critical diseases, mainly neurological diseases and decreased year by year. Second, the most common reasons for urgent consultation were disturbance of consciousness, convulsions/stiffness, weakness, and psychiatric disturbances.

The urgent inpatient neurologic consultations decreased year by year. Among the 1437 urgent inpatient neurologic consultations, the most urgent inpatient neurologic consultations were reasonable and in‐line with the predefined criteria. This reflects that the doctors in non‐neurologic departments have mastered better the relevant knowledge of these consultants diseases, which is related to the effective management of the medical department of the hospital. The medical administrators pay more attention to basic medical knowledge and skills, carrying out a large number of continuing medical education and training of residents during the study period. Since 2014, a National Standardized Training of Residents was established. Our hospital comprehensively carried out the rotation learning in the Standardized Training of Residents, and cultivated clinicians who have good professional ethics, solid medical theoretical knowledge, and clinical skills can independently standardize their work after education. For the diagnosis of common diseases in this specialty, the standardizations of physician training are realized. Second, for neurological diseases, there is a standardized clinical pathway for doctors to refer to. Non‐neurological residents should learn more about neurological disorders as well. As a result, they can make the best decision when faced with a similar situation involving urgent neurologic inpatient consultations.

In urgent consultations, acute cerebrovascular disease accounted for 33.6% of cases, including cerebral infarction, cerebral hemorrhage, and transient ischemic attack, and the average age of patients presenting to the consultations was 60.3 years, similar to previous findings (Wijdicks & Hocker, 2020), which may be related to the increase in stroke incidence with age (Rodríguez‐Antigüedad et al., 2011). Most of the consultations met the criteria for an urgent consultation because physicians are now more skilled in diagnosing and treating acute cerebrovascular disease. There were 177 cases of epilepsy/status epilepticus, which accounted for a higher proportion of consultation than the common urgent consultation diseases reported in previous research in China. The main reason may be that the study hospital has one of the famous epilepsy centers in China, which has several epilepsy specialists and a large number of regular follow‐up patients from the region and neighboring cities. Metabolic or infectious toxic encephalopathy and encephalitis cases constitute the third largest group of disorders for urgent inpatient neurologic consultations. There is a Key Laboratory of Neurology of Chinese Education Commission, which is operated by several full‐time experts. The positive results of the detection of acid‐fast bacilli are more than 80%, and abnormal cells may also be found in cerebral spinal fluid. They could also detect more than 10 antibodies associated with autoimmune encephalitis with high specificity and sensitivity. As a result, hospital neurologists can better manage the diagnosis and treatment of encephalitis as soon as possible.

Departments that frequently request urgent neurologic consultations include the ICU, respiratory medicine, cardiovascular medicine, hematology, and nephrology. In previous reports, it has been estimated that approximately 10% of patients with medical conditions will have some neurological manifestations (Coban et al., 2016; Do & Siegler, 2018; Ropper, 2016). Due to with insufficient knowledge about critical nervous system diseases, some young residents do not consult superior physicians when encountering patients with neurological diseases in the ICU, but instead invite urgent neurologic consultations, increasing consultation workload. Internal medicine residents are recommended to have at least 6 months training in neurology during the Standardized Training of Residents, with more ongoing study in critical neurology. Most of the cardiorenal patients have risk factors for vascular sclerosis and are often accompanied by cerebrovascular disease. Patients with renal failure are consulted by neurologists because of their impaired consciousness or convulsions during dialysis, which are due to internal environment dysfunction, renal encephalopathy, and dialysis encephalopathy.

Common reasons for the Department of Respiratory Medicine to apply for urgent inpatient neurologic consultations include convulsions and disturbance of consciousness, mostly due to pulmonary encephalopathy or hypoxic ischemic encephalopathy. First, it is very important for them to control the primary disease. Tuberculous meningitis should be considered if a patient with pulmonary tuberculosis presents with headache, disturbance of consciousness, convulsions, or hyponatremia, ambulatory EEG, and MRI, and a lumbar puncture should be prescribed for routine cerebrospinal fluid analysis. Once a patient is diagnosed with tuberculous meningitis, standardized treatment with antituberculosis drugs should be prescribed as soon as possible. The main reasons for requesting surgical emergency consultation are disturbance of consciousness, and uncontrolled postoperative seizure attacks.

A majority of patients with neurological conditions are admitted under the care of non‐neurological physicians (Lahlouh et al., 2020). An accurate medical history and neurological exam can help doctors differentiate between neurological disorders. A total of 150 cases of non‐neurological diseases, including agitation, mental disturbance, disturbance of consciousness, and headache. These are thought to be associated with internal environmental disturbances, primary disease, hypertension, anxiety, and vagal reflex syncope. Some doctors confuse mental disorders with aphasia and mistakenly apply for urgent neurologic consultation. Therefore, to reduce unnecessary urgent neurology consultations, strengthen clinician training is necessary. Acute encephalopathy is often secondary to infection after cardiopulmonary resuscitation or metabolic disease. It is recommended that physicians perform blood gas analysis, EEG, blood glucose and electrolyte analysis, Brain CT, and taking the medication history in detail, including sedatives, antidepressants. For postoperative intense aggravation of awareness and cognitive impairment, which frequently occurred in anesthetic drugs. Residents generally overlook it and ought to be aware of the symptoms of sedative medications.

Non‐neurological disorders accounted for 10.4% of cases. Medicine and surgery account for a half, including respiratory, cardiology, orthopedics, obstetrics, and gastroenterology. Irritability, delirium without focal neurological signs in non‐neurological disorders can be used as differential criteria for neurological disorders. Most patients with non‐neurological disease did not have new structural damage in the central nervous system (Roland et al., 2018), and the treatment was mainly to maintain the stability of the internal environment to treat the primary disease. Pain was also a common reason for counseling in previous studies (Morrish, 2015; Sakashita et al., 2018). There are cases in which a neurosurgeon should be consulted rather than a neurologist, such as brain contusions and lacerations, basilar fractures, diffuse axonal injury, intracranial tumors, hydrocephalus, and subdural hematoma. Therefore, it is necessary for clinicians to have a basic knowledge of neurology and neurosurgery.

In addition, some consultations should be regularly rather than urgent. For example, a patient with Parkinson's disease requested an urgent inpatient neurologic consultation for an intravenous ganglioside infusion. Preoperative evaluation and secondary prevention of cerebrovascular disease are performed surgically sometime, and it is better to request regular neurologic inpatient consultations rather than urgent inpatient neurologic consultations.

## 5 CONCLUSIONS

Non‐neurological medical staff should strengthen their understanding of common acute and critical illnesses, and neurologists should provide more on‐the‐job training related to neurological diseases for young doctors in the hospital to reduce blind spots and inappropriate clinical work. For invitation to urgent inpatient neurologic consultation, the Standardized Training of Residents is helpful to rapidly improve the comprehensive diagnosis and treatment ability of young residents and is suitable for application in hospitals at all levels.

### 5.1 Limitations

This study examined all urgent inpatient neurologic consultations at the hospital over a 4‐year period. Although this study is the first time to conduct a large‐scale retrospective sampling survey of inpatients applying for urgent inpatient neurologic consultations, due to the limitation of the distribution of disciplines in the hospital, there are still 169 cases of undiagnosed causes in the consultation application due to serious illness, incomplete relevant examinations, or poor physician's records. In order to fully grasp the current situation of urgent inpatient neurologic consultations, a large‐sample, multicenter study should be carried out in the future.

## AUTHOR CONTRIBUTIONS

Jiafang Wang and Min Ren contributed to the study conception and design. Data collection was performed by Jiafang Wang, Hong Wang, Zhengzhen Bai and Kebin Zeng. Statistical analysis was performed by Jiafang Wang, Hong Wang, Zhengzhen Bai, and Kebin Zeng. The first draft of the manuscript was written by Jiafang Wang. Min Ren and Kebin Zeng commented on previous versions of the manuscript. All authors read and approved the final manuscript.

## CONFLICT OF INTEREST STATEMENT

The authors declare that the research was conducted in the absence of any commercial or financial relationships that could be construed as a potential conflict of interest.

### INFORMED CONSENT

Written consent was waived, as this retrospective study does not influence the health care of included individuals. All patients’ data were anonymized.

### PEER REVIEW

The peer review history for this article is available at https://publons.com/publon/10.1002/brb3.2983.

## Data Availability

Data would be available by contacting corresponding author and after excluding the personal information of patients.
